# Insights about genome function from spatial organization of the genome

**DOI:** 10.1186/s40246-018-0140-z

**Published:** 2018-02-20

**Authors:** Shuvra Shekhar Roy, Ananda Kishore Mukherjee, Shantanu Chowdhury

**Affiliations:** 1 Genomics and Molecular Medicine Unit, CSIR-Institute of Genomics and Integrative Biology, Mathura Road, New Delhi, 110025 India; 2 Academy of Scientific and Innovative Research, CSIR-Institute of Genomics and Integrative Biology, Mathura Road, New Delhi, 110025 India

**Keywords:** Genome architecture, Chromosome conformation capture (3C), Hi-C, Topologically associated domains (TAD), Chromatin looping, Histone modifications, Transcription

## Abstract

Over the last 15 years, development of chromosome conformation capture (3C) and its subsequent high-throughput variants in conjunction with the fast development of sequencing technology has allowed investigators to generate large volumes of data giving insights into the spatial three-dimensional (3D) architecture of the genome. This huge data has been analyzed and validated using various statistical, mathematical, genomics, and biophysical tools in order to examine the chromosomal interaction patterns, understand the organization of the chromosome, and find out functional implications of the interactions. This review summarizes the data generated by several large-scale high-throughput chromosome conformation capture studies and the functional implications obtained from the data analyses. We also discuss emerging results on factors (both CCCTC binding factor (CTCF) related and CTCF independent) that could contribute to looping interactions.

## Background

Recent advances in the field of *c*hromosome *c*onformation *c*apture (3C) and its subsequent advancements like 4C (chromosome conformation capture-on-chip), 5C (carbon copy chromosome conformation capture), and high-throughput chromosome conformation capture (Hi-C) have greatly expanded the general understanding of the three-dimensional spatial arrangement of the genome. Consequently, the notion of a linear chromosomal distribution of various elements of the coding and the non-coding genome is being revisited. This has brought forth a new perspective on how genomic functions such as replication, transcription, and DNA repair would be investigated. Interestingly, the proximity of various regions of the genome, which were otherwise unintuitive, now opens new possibilities by which various aspects of gene regulation would be studied in future. Along with these, herein, we also discuss recent data suggesting the role of telomeres in looping interactions, both near and with interstitial regions of the genome. In addition, factors other than the chromatin-associated protein CCCTC binding factor (CTCF) that could mechanistically contribute to the formation of chromatin loops are also discussed.

## The spatial organization of the genome from Hi-C data

### All to all chromosome interaction matrices reveal compartments within genomes

Hi-C is a high-throughput NGS (next-generation sequencing)-based version of chromosome conformation capture assay commonly known as 3C [1]. It provides a global view of all chromosomal interactions across the genome. In such experiments, tagged nucleotides are used to capture ligated products after restriction digestion and appropriately diluted ligation. This is followed by sequencing of the captured DNA fragments. The resulting reads represent ligation of fragments both adjacent to each other as well as far apart in the linear genome. A genome-wide two-dimensional contact matrix is generated by dividing the genome or each chromosome into bins of equal distance and then aligning these reads into those bins. Such matrices depict the collective average of the interactions present within the population of cells used for Hi-C studies [2]. There are several other 3C-based assays to look at chromosomal interactions, but the advantage of Hi-C experiments is that they give a global view of all the chromosomal interactions across the genome.

Though we obtain vast knowledge on the biology of chromosomal interactions and genome organization from Hi-C data, there are some drawbacks of the method as well. The Hi-C data, for instance, does not provide much quantitative information on the stability and strength of chromosomal interactions. Most of the reported Hi-C studies so far used asynchronized cells and did not consider the variability that the different cell cycle stages (particularly mitotic phase) would most likely introduce into the chromatin conformation. Several reviews have further described the technical aspects of these 3C-based assays and complimentary microscopy techniques [3–5].

In 2009, Lieberman-Aiden et al. used karyotypically normal GM06990 human lymphoblastoid cells to generate 8.4 million reads that uniquely aligned to the human genome reference sequence in a Hi-C experiment; of these, 6.7 million reads corresponded to long-range contacts between segments of the genome > 20 Kb apart. The long-range contact reads were used to construct genome-wide contact matrices by dividing the genome into 1-Mb-sized bins. This revealed an interesting pattern comprising distinct intra- and inter-chromosomal genomic compartments with contacts that were primarily restricted within compartments [2].

A few years later, using Hi-C contact matrices of bin sizes ranging from 20 to 100 Kb, about 1.7 billion read-pairs of data were analyzed and genome-wide formation of compartments or topologically associated domains (TADs) in mouse and human-differentiated and embryonic stem (ES) cells was reported [6]. This was supported by work from Jin et al. who studied chromatin interactions in response to transient TNF-alpha signaling in IMR90 primary human fibroblasts by performing Hi-C before and after 1 h TNF-alpha treatment [7]. This gave about 3.4 billion uniquely mapped paired-end reads from multiple biological replicates, among which, approximately 1.4 billion were intra-chromosomal reads.

**Fig. 1:**
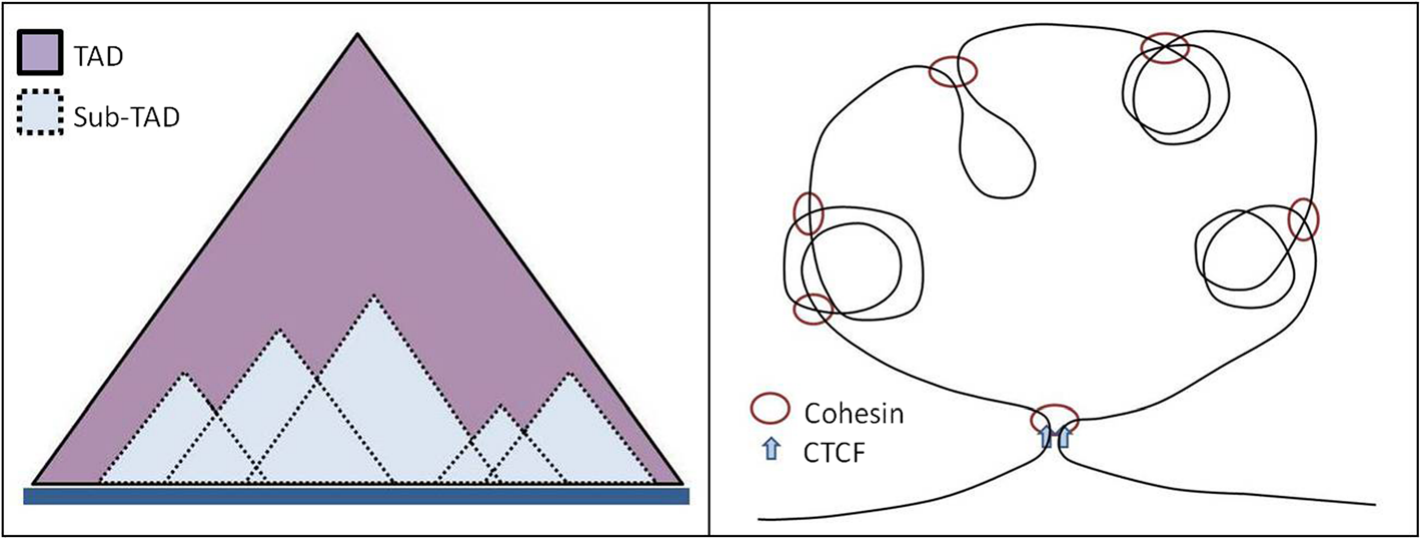
Schematic representation of how smaller TAD-like structures (sub-TADs) emerge from Hi-C contact matrix and how they might be forming in three dimensions. Dynamic loop extrusion by factors like Cohesin might lead to looping and higher interactions in the extruded loci leading to emergence of sub-TADs

A slightly different approach termed as in situ Hi-C was used by Rao et al. where DNA-DNA proximity ligation was carried out inside intact nuclei—in situ ligation reduced the chances of spurious contacts due to random ligation in dilute solution [8]. This also yielded a higher resolution of up to 1 Kb bin size and 200- to 1000-fold more contacts. Using the high-resolution contact matrices, it was possible to see many smaller contact domains (sub-TADs) with higher intra-domain contact frequency like the topologically associated domains and other similar small domains discussed in several Hi-C studies (Fig. 1) [6, 9, 10].

### Contacts prevalent within chromosomes rather than across chromosomes

**Fig. 2:**
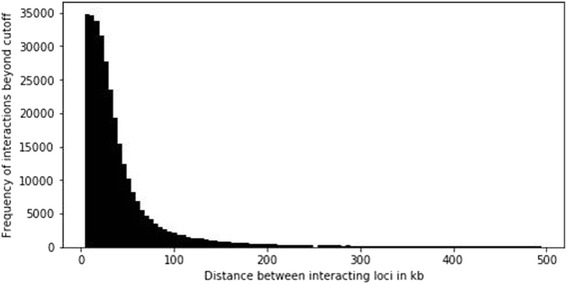
Frequency distribution of interactions (above cutoff of 10 units of interaction value for a pair of loci) with distance between the interacting loci by analyzing intra-chromosomal Hi-C contact matrix of human chromosome 5 (analysis was done using normalized interaction values from Hi-C data given in Rao et al. [8])

Chromosomal interactions across the whole genome noted that the average intra-chromosomal contact probability between pairs of loci in a chromosome decreased consistently with increasing genomic distance (Fig. 2) [2]. Interestingly, this suggested polymer-like behavior of the genome where the three-dimensional distance between pairs of loci increases with increasing genomic distance. Other studies on chromosomal organization via 3C and fluorescence in situ hybridization (FISH) also made similar observations [1, 11]. Even at distances greater than 200 Mb, the average intra-chromosomal contact probability was always more than the average contact probability between different chromosomes (inter-chromosomal), implying the existence of chromosomal territories.

Intriguingly, probabilities of inter-chromosomal contacts show that small, gene-rich chromosomes (chromosomes 16, 17, 19, 20, 21, and 22) preferentially interact with each other. Furthermore, FISH studies also observed these chromosomes to frequently localize to the center of the nucleus [12, 13]. On the other hand, another small but gene-poor chromosome, i.e., 18, was found to interact less with other chromosomes, and FISH studies showed chromosome 18 tends to be located near the nuclear periphery [14].

### Genome topologies constructed from interaction matrices

Using principal component analysis, each chromosome could be partitioned into two compartments (termed A and B) such that loci within the same compartment had correlated contact profiles. Through this correlation, even loci belonging to different chromosomes were assigned the same compartment, resulting in the whole genome being divided into two spatial compartments such that greater interactions occurred within than across compartments [15].

It was observed that loci in compartment B had a higher tendency for close spatial localization, suggesting a relatively compact state of chromatin. On the other hand, loci within compartment A showed significant correlation with the presence of genes, higher expression, and chromatin accessibility (DNase I hypersensitivity) and were enriched for activating chromatin marks (H3K36me3). Thus, compartment A could be associated with open, accessible, and actively transcribed chromatin. Together, these suggested open and closed chromatin domains throughout the genome occupy different spatial compartments in the nucleus.

At higher resolution (bin sizes less than 100 Kb), highly self-interacting regions were found to emerge, seen as triangles in the interaction matrix heat map [6]. These regions were termed as topological domains. Topological domains were found to be bound by narrow segments where the chromatin interactions appear to end abruptly. Using a statistic termed directionality index (DI), authors identified 2200 topological domains in mouse ES cells with a median size of 880 Kb that covered ~ 91% of the genome. DI models the difference between a number of upstream and downstream interactions at a given locus along a chromosome, and thereby boundaries of topological domains were detected where there was a significant shift from contact points oriented with upstream vis-à-vis downstream bias. Also, as expected, the frequency of intra-domain interactions was noted to be higher than that of inter-domain interactions.

### Boundary demarcations between distinct genome topologies

Consistent with this, fluorescent in situ hybridization (FISH) experiments revealed pairs of loci within a particular topological domain were closer in space than pairs of loci in different topological domains in spite of similar genomic distances between the loci [16]. The genomic regions between the topological domains were defined as either “topological boundary regions” or “unorganized chromatin,” depending on their sizes (topological boundary regions: median ~ 0 Kb, 76.3% of the regions < 50 Kb; or unorganized chromatin: median ~ 560 Kb). Moreover, the topological domains correlated with other described components of the genome-like compartments A and B [2], replication time zones [17, 18], and large organized chromatin K9 modification (LOCK) domains [19]. A large subset of the identified domain boundaries also appeared to mark the transition between LAD (lamina-associated domain) and non-LAD regions in the genome [20, 21].

An in situ Hi-C study using high-resolution contact matrices revealed numerous relatively small contact domains with higher intra-domain contact frequency [8] like topologically associated domains or other small domains discussed in other Hi-C studies [6, 9, 10].

Interestingly, the in situ Hi-C data revealed six nuclear sub-compartments based on long-range interaction patterns, both intra-chromosomal and inter-chromosomal, using different approaches to clustering. On comparison with compartment A/B [2], two of the six interaction patterns correlated with loci in compartment A—termed as sub-compartments A1 and A2. These loci were gene dense, harbored highly expressed genes, enriched in activating chromatin marks, and were depleted at the nuclear lamina- and nucleolus-associated domains (NADs). Rest of interaction patterns correlated with loci in compartment B with very different properties than A1 and A2.

The DI data used to identify TADs can vary substantially depending on the sliding window size selected with small window sizes giving smaller TADs and larger ones yielding larger TADs which often nest groups of smaller domains [22]. In fact, reanalysis of the original Hi-C data from which megabase-sized TADs were identified [6] with a different algorithm using smaller window sizes led to the observation that larger conserved TADs tend to consist entirely of smaller domains. These domains were found to be stable across cell lines and persistent across resolutions, with their boundaries having high enrichment in CTCF binding and activating histone marks [22].

### Genome compartments: fractal globule versus topologically associated domain architecture

Chromosomal regions have been perceived as an “equilibrium globule”—a compact and densely knotted configuration originally used to describe a polymer in a poor solvent at equilibrium [23, 24]. An alternative model proposes that polymers, including interphase DNA, can self-organize into a long-lived, non-equilibrium conformation described as “fractal globule” [25, 26]. This dense, compact state is adopted by an untangled polymer as it crumbles into a series of small globules in a “beads-on-a-string” configuration. These beads act as monomers in further rounds of spontaneous crumpling until only a single globule of globules of globules remain.

Lieberman-Aiden et al. analyzed the scaling of contact probability of fractal globule and found that it is close to the contact probability observed from the Hi-C data [2]. The predicted scaling of three-dimensional (3D) distance between pairs of loci based on the fractal globule model is also close to the scaling reported by 3D FISH for genomic distances between 500 Kb and 2 Mb [24]. At a scale of several megabases, the data is consistent with a fractal globule model for chromatin organization. Fractal globule is an attractive model to define chromatin organization since they are free of knots [27] and in principle consistent with unfolding and refolding, for instance, during gene regulatory events like activation and repression or processes such as replication and recombination.

## Functional implications of domain formation in the genome

### Domain boundaries associated with gene promoters and transcription

**Fig. 3:**
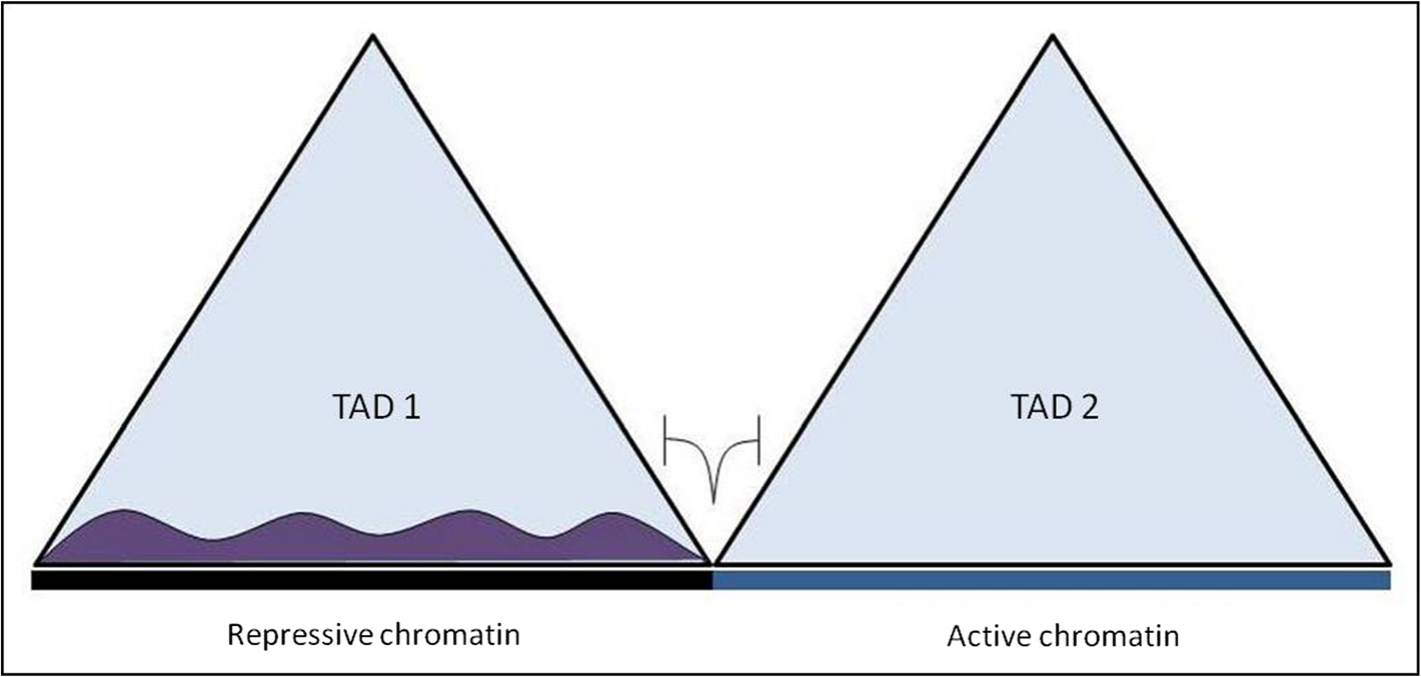
Schematic representation to show that TAD boundary restricts the spread of heterochromatinization

A strong enrichment of CTCF binding sites was observed at the TAD boundary regions [6], a property also common with many known insulator or barrier elements [20, 28, 29]. Like a classical boundary element is known to stop the spread of heterochromatin, a clear segregation of the heterochromatin marker H3K9me3 modification was observed within the TAD boundary regions (Fig. 3).

Several studies found TADs to be majorly conserved across cell types in a given organism, suggesting TADs to be stable structures of the *3D* genome organization [6, 9, 30]. On the other hand, the smaller scale structures, like sub-TADs, loops, and insulation neighborhoods, all show at least partial variation between different cell lineages, with the variations in their organization appearing to be related with cell type [8, 31–33]. The dynamic chromatin interactions varying across cell types were also enriched for differentially expressed genes [6, 34, 35].

These observations suggest that chromatin organization as TADs is mostly stable across cell types, within which specific structures and dynamic interactions can form to play lineage and context-specific regulatory roles contributing to molecular events associated with differentiation [36].

Interestingly, the topological domains do not seem to be the consequence of heterochromatin formation as the detected boundaries were present in both pluripotent cells and their differentiated progeny, i.e., before and after heterochromatinization associated with cellular differentiation. Importantly, this implied that the topological domains along with boundaries delineate the endpoints of heterochromatic spreading [6].

Furthermore, enrichment of chromatin marks associated with promoters and gene bodies and depletion of repressive chromatin marks were detected in the TAD boundaries along with enrichment of housekeeping genes, transcription start sites, and global run on sequencing signal in the boundaries. Together, these suggest a high level of transcriptional activity associated with boundary formation; however, whether boundary formation was a cause or consequence of transcriptional activity was not clear [6].

### Point-to-point direct interactions—looping of chromatin affects gene transcription

Interestingly, Hi-C data also revealed pairs of loci that had significantly stronger interaction than any loci lying between them. These were designated as loops, and can appear within topology domains (discussed above), independently and/or across TADs. Interestingly, chromatin loops were not only conserved among human cell lines but also found to be conserved between mouse and human cells [8]; chromatin looping interactions were significantly enriched within cis-regulatory elements like active promoters and enhancers while being depleted at inactive TSS or regions with repressive chromatin marks [6–8]. About 30% of chromatin loops brought promoters and enhancers together (versus 7% expected by chance), and genes with promoters associated with loops were more expressed than genes whose promoters were not associated with any loop (sixfold) [8]. Hi-C data analysis also revealed 55% of distal enhancers interact with at least one active promoter, confirming previous observations that promoters and enhancers often form complex networks to regulate transcription [7, 37]. Interestingly, a particular case study showed many genes without any NF-kappaB (p65) binding site in promoters were induced simultaneously by TNF-alpha (which is known to trigger NF-kappaB signaling), possibly due to sharing of overlapping distal interacting regions containing multiple NF-kappaB binding sites [7].

Somewhat intriguingly, there was little or no change in promoter-enhancer looping interactions at a vast majority of TNF-alpha-responsive enhancers on TNF-alpha treatment. This suggested that in general, promoter-enhancer contacts in untreated cells, which are the existing DNA loops, did not alter upon transient activation or repression of enhancers following treatment. Interestingly, chromatin interactions involving cell type-specific enhancers are variable between cell types indicating context-specific interaction structures. This discrepancy between signal-dependent and cell type-specific enhancers correlates with H3K4me1 chromatin marks, which unlike H3K27me3 remain unchanged upon TNF-alpha treatment [7]. Other studies have also observed pre-existing looping interactions at several loci induced by p53, FOXO3, and glucocorticoid receptors [33, 38, 39].

### Chromatin loops marked through CTCF binding

Analysis of ENCODE ChIP-seq data revealed loci with chromatin loops were typically bound by the insulator protein CTCF (86%) and the Cohesin subunits RAD21 (86%) and SMC3 (87%) [8]. This was consistent with many reports which, using a variety of experimental approaches, suggest a role for CTCF and cohesion in mediating DNA loops [28, 40, 41]. As many of these loops demarcate domains, this observation was also concordant with studies showing CTCF delimits structural and regulatory domains [6, 42, 43]. Furthermore, most peak loci possessed a unique DNA site containing a CTCF binding motif to which all the three proteins (CTCF, Rad21, and SMC3) bind. A vast majority of these motif pairs present in the peak loci were oriented in a convergent manner suggesting that a pair of CTCF motifs in the convergent orientation might be required for the formation of a loop [8].

### Looping through telomere ends

Studies by Shay and Wright’s groups have demonstrated that telomeres loop to specific loci (within 10 Mb)—a phenomenon called TPE-OLD (telomere positioning effect—over long distances). These studies showed that genes close to the telomere were silenced in young primary cells with long telomeres, but were activated when telomeres became short with cellular aging, an effect that was reversed by re-elongation of telomeres upon exogenous expression of the hTERT (human telomerase) gene. Interactions of the telomere and sub-telomere with chromosomal regions up to 10 Mb away from the telomere end were revealed using modified Hi-C and 3C experiments [44–47]. An important function of such looping showed looping of chromosome 5 sub-telomere resulted in heterochromatic silencing of the telomerase gene in young primary cells [44]. Reports also suggest that several telomere binding proteins like TRF2 and TIN2 can associate with interstitial telomere-like repeats [48]. Such regions of repetitive DNA (often referred to as interstitial telomeric repeats or ITS) may be crucial in forming sub-telomeric loops by recruitment of telomeric factors and structural proteins like Lamins to mediate interaction with telomeres [49, 50].

### Single cell Hi-C

Though Hi-C has given insights into the functional chromosomal organization, one can argue that this is an average, probabilistic view of chromosomal interactions with much cell-to-cell variation, such that observed domain organization and chromosomal interactions might represent just a fraction of the cells. Some recent studies attempt to address this with Hi-C at the single cell level. In the first such report, pooled data recapitulated the formation of TAD-like structures indicating these domains are robust and form the basis of chromosome conformation in each cell [51]. However, the observed variability in inter-domain contacts suggested significant differences might be possible in the higher order folding of chromosomes. Another single cell study concluded that, though structures of individual TADs and chromosome loops vary substantially from cell to cell, the higher order organizational signatures like the A/B compartments are mostly retained [15, 52]. In addition, it was also noted that LADs and active enhancers and promoters are consistently organized genome-wide in every cell [52].

## Other factors that nucleate chromatin interactions in the 3D genome

The finding that a large fraction of loop boundaries are bound by CTCF and the Cohesin subunits [8] led to popularization of the extrusion model of loop formation. In this model, a pair of factors (Cohesin), possibly with motor function, can dynamically bind to DNA and move along the DNA in opposite directions extruding the chromatin to form loops in between until they dissociate or stall at a boundary element (CTCF motif) [53–55]. This model largely helps to understand the nested nature of TADs and loops as well as the consequences of CTCF motif deletion and inversion [36, 56]. However, many convergent CTCF motifs exist that do not delineate loops and, interestingly, a considerable fraction of identified loops did not appear to possess any CTCF or cohesion binding sites. Together, these suggest other factors that could be involved in loop formation.

The fact that CTCF motifs can act as boundary elements only when they are oriented in convergent fashion indicate dimerization of CTCF is required for stalling extruding cohesion loops. This indicates that interactions between DNA binding proteins can possibly play a role in mediating loop formation and chromatin interactions by bringing distant genomic loci together. Indeed, DNA binding proteins, YY1 and ZNF143, were found to be enriched in loop loci [8]. Both homodimer and heterodimer formation by proteins bound to distant loci can lead to chromosomal looping interactions. In a somewhat similar context, the telomere binding factor TRF2 has already been implicated in mediating telomeric looping into the extra-telomeric interstitial regions including the TERT loci [44]. The fact that TRF2 can bind to many extra-telomeric sites throughout the human genome implicates possibility of it mediating other looping interactions as well [49, 50, 57, 58].

Apart from proteins, nucleic acids like lncRNAs and secondary DNA structures might also contribute to mediating chromosomal looping interactions. lncRNAs called as activating/enhancer RNAs were found to bind with mediator proteins (MED12 and MED1), and their knockdown led to diminished looping between the ncRNA loci and their target genes and a decrease in mediator binding to the genes [59, 60].

Another interesting context that could support loop formation in a CTCF-independent way comes from the possible involvement of DNA secondary structures such as G quadruplexes. G quadruplexes are typically formed by a stretch of DNA with 3-guanines repeated at least four times at close interval and are reported to be involved in various biological functions across life forms [61–64]. The formation of inter-molecular G quadruplexes raises the possibility that such secondary structure DNA motifs can bring together two distant genomic loci. G quadruplexes have been found to be enriched in DNAse hypersensitive (DHS) promoters as well as DHS cis-regulatory elements [65, 66]. Half G quadruplexes (i.e., two runs of guanines, both containing at least three consecutive Gs) were also found to be enriched at the boundaries of these DHS promoters and *cis*-elements but were depleted in the vicinity of these sites. Computational analyses showed such half G quadruplexes, one from DHS promoter and one from DHS enhancer, could come together to form G quadruplexes thereby bringing together promoters and enhancers elements via looping [65]. Several studies have reported enrichment of potential G quadruplex forming sequences in fragile genomic regions associated with pathogenic, cancer-causing breakpoints, and structural variations [67–70]. The possible formation of chromosomal loops mediated by G quadruplexes could be one of the contributing factors in increasing the fragility of such chromosomal regions. Disruption of these DNA structure motifs could give a clear idea regarding their roles in mediating functional chromosomal interactions.

## Disruption of TAD boundaries has deleterious effects: clinical implications

**Fig. 4:**
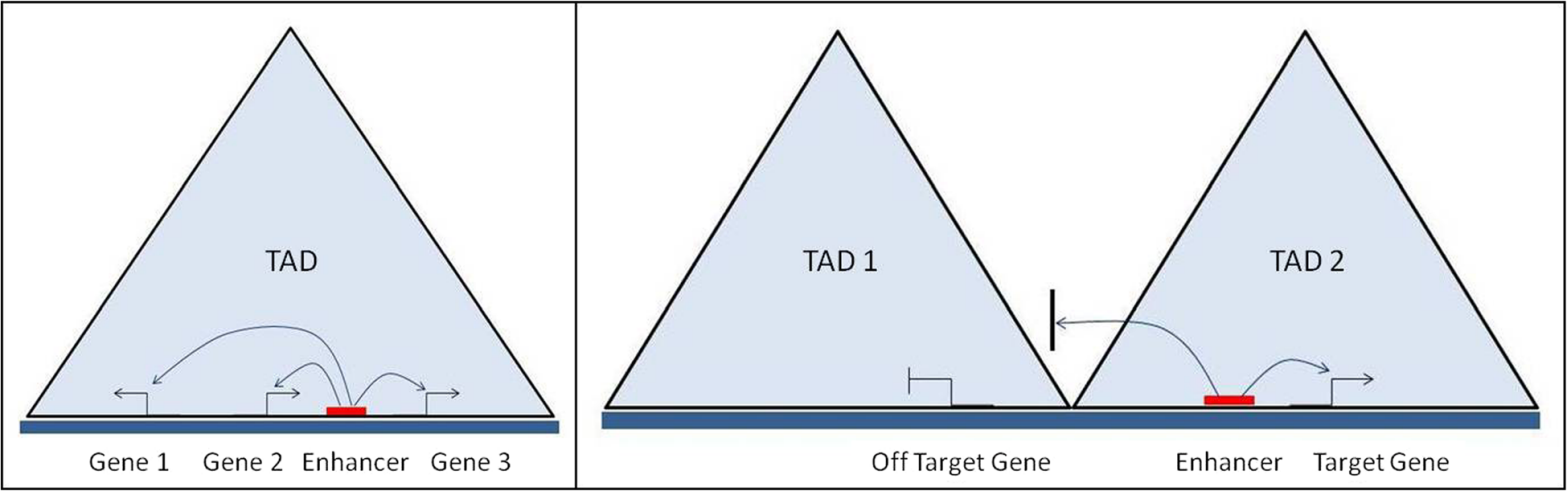
Schematic representation showing coordinated expression of genes mediated by an enhancer interacting with multiple promoters within the same TAD and a TAD boundary restricts enhancer interaction and activity only to target genes within the same TAD

Deletion of a TAD boundary, on the other hand, was found to result in spreading of contacts across the deleted region and transcriptional misregulation [9]. Several reports of TAD disruptions leading to pathological outcomes have further consolidated TADs as functional units. Structural variants disrupting CTCF-associated TAD boundaries were noted to allow de novo promoter-enhancer interactions and ectopic gene expression causing limb malformation [71] and oncogenic transformation [72]. Oncogenic chromosomal rearrangements were also shown to induce aberrant oncogenic expression in AML and medulloblastoma by causing enhancer trafficking where an enhancer acts on a gene other than its normal target due to genomic rearrangements like TAD disruptions [73, 74]. Moreover, it was also reported from Hi-C in prostate cancer cells that generation of smaller TADs due to establishment of additional boundaries and particular cancer-specific interactions within TADs could be associated with oncogenic transformation [75], and perturbation of CTCF binding by aberrant DNA methylation could cause oncogenic gene expression leading to gliomas by a mechanism similar to “enhancer hijacking” [76]. These pathogenic outcomes based on deregulated enhancer function along with another reporter construct insertion study [77] implicates that cis-regulatory elements can act in a non-specific manner but within a given TAD in the genome (Fig. 4).

Apart from how abrogation of TADs could lead to pathogenic phenotypes, chromatin looping interactions, even when not associated with a particular TAD, might have significant clinical implications. Recently, an interaction has been reported that might be crucial for TERT reactivation across different cancer types. The two cytosine to thymidine single-point mutations in the TERT proximal promoter (− 146 and − 124 bp from the translation start site), recurrent in several cancers, were found to create a de novo consensus binding motif for ETS transcription factor family [78–83]. GABPA (an ETS transcription factor) was shown to bind to the mutant promoter leading to a long-range chromatin interaction with a region 300 Kb upstream of the TERT promoter. As an effect of this binding, the promoter locus was changed into an open, active chromatin region, eventually enhancing TERT expression which has been widely associated with cancer development [84–86]. In another study, it was shown that telomere looping at the 4q35 locus regulates expression of SORBS2, which is disrupted in the age-associated genetic disease facioscapulohumeral muscular dystrophy [45].

## Conclusion

Many large-scale studies with vast Hi-C data have given us important insights into possible mechanisms of looping, subsequent higher order chromatin organization, and functional significance of domain formation and chromosomal interactions in different processes including transcriptional regulation. Given that these observations encourage a shift in perspective regarding how *3D* organization of the genome impacts biological processes, it is of much interest to understand the underlying mechanistic rules governing causation of the folded architecture. Though cohesion and CTCF have been implicated in the formation of chromosomal loops, these proteins are absent in a fraction of loop loci (~ 10–14%) suggesting a role of other factors in determining how chromosomal interactions arise and how these contribute to specific cellular and context-specific functions.

## Abbreviations

3C: *C*hromosome *c*onformation *c*apture
3D: Three-dimensional
4C: Chromosome conformation capture-o-chip
5C: Carbon copy chromosome conformation capture
AML: Acute myeloid leukemia
ChIP-seq: Chromatin immunoprecipitation-sequencing
CTCF: CCCTC binding factor
DI: Directionality index
ENCODE: Encyclopedia of DNA elements
ES: Embryonic stem cells
FISH: Fluorescence in situ hybridization
hTERT: Human telomerase reverse transcriptase
LAD: Lamina-associated domains
lncRNA: Long non-coding RNA
LOCK: Large organized chromatin K9 modifications
NAD: Nucleolus-associated domains
NGS: Next-generation sequencing
TAD: Topologically associated domain
TIN2: TERF1-interacting nuclear factor 2
TNF: Tumor necrosis factor
TPE-OLD: Telomere position effect-over long distances
TRF2: Telomere repeat binding factor 2
TSS: Transcription start site
